# Age-Related Sexual Dimorphism on the Longitudinal Progression of Blood Immune Cells in BALB/cByJ Mice

**DOI:** 10.1093/gerona/glab330

**Published:** 2021-11-06

**Authors:** Cláudia Serre-Miranda, Susana Roque, Palmira Barreira-Silva, Claudia Nobrega, Neide Vieira, Patrício Costa, Joana Almeida Palha, Margarida Correia-Neves

**Affiliations:** Life and Health Sciences Research Institute, School of Medicine, University of Minho, Braga, Portugal; ICVS/3B’s—PT Government Associate Laboratory, Braga/Guimarães, Portugal; Life and Health Sciences Research Institute, School of Medicine, University of Minho, Braga, Portugal; ICVS/3B’s—PT Government Associate Laboratory, Braga/Guimarães, Portugal; Life and Health Sciences Research Institute, School of Medicine, University of Minho, Braga, Portugal; ICVS/3B’s—PT Government Associate Laboratory, Braga/Guimarães, Portugal; Life and Health Sciences Research Institute, School of Medicine, University of Minho, Braga, Portugal; ICVS/3B’s—PT Government Associate Laboratory, Braga/Guimarães, Portugal; Life and Health Sciences Research Institute, School of Medicine, University of Minho, Braga, Portugal; ICVS/3B’s—PT Government Associate Laboratory, Braga/Guimarães, Portugal; Life and Health Sciences Research Institute, School of Medicine, University of Minho, Braga, Portugal; ICVS/3B’s—PT Government Associate Laboratory, Braga/Guimarães, Portugal; Life and Health Sciences Research Institute, School of Medicine, University of Minho, Braga, Portugal; ICVS/3B’s—PT Government Associate Laboratory, Braga/Guimarães, Portugal; Life and Health Sciences Research Institute, School of Medicine, University of Minho, Braga, Portugal; ICVS/3B’s—PT Government Associate Laboratory, Braga/Guimarães, Portugal

**Keywords:** Adaptive immune, system, Aging, Animal model, Blood, Gender differences, Immunosenescence, Innate immune system, Linear mixed models, Sex

## Abstract

The study of immune system aging is of relevance, considering its myriad of interactions and role in protecting and maintaining body homeostasis. While mouse models have been extensively used to study immune system aging, little is known on how the main immune populations progress over time and what is the impact of sex. To contribute to filling this gap, male and female BALB/cByJ mice were longitudinally evaluated, from 3 to 18 months old, for the main blood populations, assessed by flow cytometry. Using linear mixed-effect models, we observed that the percentages of neutrophils, monocytes, eosinophils, and total natural killer (NK) cells increase with aging, while those of B cells, T cells (including CD4^+^ and CD8^+^ subsets), and Ly6C^+^ NK cells decrease. Males present higher percentages of neutrophils and classical monocytes Ly6C^high^ over time, while females present higher percentages of total T cells, both CD4^+^ and CD8^+^, eosinophils, and NK cells. Males and females display similar percentages of B cells, even though with opposite accelerated progressions over time. This study revealed that mouse models recapitulate what is observed in humans during aging: an overall proportional decrease in the adaptive and an increase in the innate immune cells. Additionally, it uncovers an age-related sexual dimorphism in the proportion of immune cells in circulation, further strengthening the need to explore the impact of sex when addressing immune system aging using mouse models.

The increasingly aged population, and its inherent association with health decline, challenges the scientific community to better understand the physiology of healthy aging. The immune system is one of the central body systems, given its own functions and the myriad of interactions it establishes with other organ systems. Therefore, it is relevant to understand how the immune system ages, influences, and is influenced by aging.

The immune system senescence, also known as immunosenescence, is characterized by progressive alterations in the proportion and function of immune cells throughout aging. In the innate immune system aging, while changes in cell function (eg, reduction in antigen presentation and in phagocytic capacity) have been consistently reported, studies exploring the numbers and frequency of cells mainly in humans often present inconsistent findings (1–4). With respect to the adaptive immune system, studies mostly in humans showed that a characteristic feature of aging is the reduction in the number of T and B cells, which is accompanied by a shrinking of their repertoire diversity, an increase of memory and activated cells, and a reduction in naïve cells (5–7). However, a comprehensive characterization of the age-related cellular characterization is poorly studied in rodents.

The cumulative alterations of the immune system over time ultimately lead to increased susceptibility to infection and reduced responsiveness to vaccination (8,9). Of interest, men and women experience those alterations differently, being the immune response typically stronger in women than in men (9,10). In other species (eg, other mammals, insects, and reptiles), a similar trend is observed regarding the immune response sexual dimorphism (9). In addition, human studies have further denoted a sexual dimorphism in age-related changes of blood cellular composition; however, information on animal models, especially rodents, is still scarce (11–14).

The mouse model has been unique to understanding human physiology in health and disease. Most published reports studying immunosenescence compare young versus aged mice, with a cross-sectional experimental design (15–17), missing therefore the opportunity to study inter- and intravariability in the aging processes. To contribute in filling this gap, in the present study, we investigated the individual progressions of the main blood adaptive and innate immune cell populations and whether those were sex-dependent in mice.

## Method

### Animals

Male and female BALB/cByJ mice, 6 weeks old, were purchased from Charles River and maintained until 18 months of age at the Life and Health Sciences Research Institute animal facility. Mice were initially housed in groups of 5 per cage under standard laboratory conditions (light/dark cycle of 12/12 hours; 22°C; 55% humidity), food and water ad libitum. Males were regrouped whenever aggressive behaviors were observed. Health monitoring was performed according to FELASA guidelines, confirming the Specified Pathogen-Free health status of sentinel animals maintained in the same animal room. Mice well-being was routinely evaluated: Animals were euthanized whenever open wounds from aggressions, visible tumors, or over 20% weight loss (in relation to the highest weight achieved) were observed. Blood samples were collected from the same animal at approximately 3, 6, 10, 12, 15, and 18 months of age.

Three independent experiments (referred as set 1, set 2, and set 3) were performed, and the number of mice used is listed in Supplementary Table 1. A total sample size of 218 mice (approximately 35 mice per sex/per experiment/set) was used. Sample size was previously determined using G*Power 3.1.2 (18) assuming an analysis of variance for repeated measures, a medium effect size [(*f*(*V*) = 0.25)], an alpha of 0.05, a statistical power of 0.8, and 2 groups with 6 measurements.

At the end of the study, mice were anesthetized with a mixture of ketamine (75 mg/kg, intraperitoneally [i.p.]) and medetomidine (1 mg/kg, i.p.) and were transcardially perfused with 0.9% saline. All procedures were carried out following European Regulations (European Union Directive 2010/63/EU). The animal facility and people directly involved in animal experimentation were certified by the Portuguese regulatory entity—*Direção Geral de Alimentação e Veterinária* (DGAV). All experiments were approved by the Ethics Committee of the University of Minho and by the national competent entity DGAV (#009458).

### Flow Cytometry

Peripheral blood was collected through a small incision on the tail to heparin-coated capillaries. Fifty microliters of blood were incubated for 20 minutes in the dark, at room temperature (RT), with the following combination of previously titrated antibodies (all from BioLegend, San Diego, CA, USA): anti-mouse CD49b FITC (clone: DX5); anti-mouse CD62L PE (clone: MEL-14); anti-mouse CD19 PercpCy5.5 (clone: 6D5); anti-mouse CD3 PECy7 or APC (clone: 145-2C11); anti-mouse CD44 APC or BV605 (clone: IM7); anti-mouse CD4 BV421 (clone: RM4-5); anti-mouse CD8 BV510 (clone: 53-6.7); anti-mouse Ly6G BV650 (clone: 1A8); anti-mouse Ly6C BV711 (clone: HK1.4); and anti-mouse CD45.2 BV785 or PECy7 (clone: 104). Erythrocytes were lysed with ammonium–chloride–potassium buffer (0. 15 M NH_4_Cl, 10 mM KHCO_3_, 0.1 mM Na_2_ EDTA) for 10 minutes in the dark, RT. Samples were washed twice with fluorescence-activated cell sorting (FACS) buffer (phosphate-buffered saline with 0.5% bovine serum albumin and 0.01% sodium azide) and centrifuged at 300 ×*g* for 5 minutes, RT. Samples were acquired (minimum of 100 000 CD45.2^+^ events/sample) on a BD LSRII flow cytometer using the FACS DIVA software (Becton and Dickinson, Franklin Lakes, NJ, USA). Samples from each set timepoint were acquired in the same run. Data were analyzed using FlowJo software (Becton and Dickinson), and the different immune cell populations were defined as follows: eosinophils (CD45.2^+^ Ly6C^−^ SSC-A^high^), NK cells (CD45.2^+^ CD49b^+^; negative for CD4, CD8, CD19, and Ly6G; subcategorized as Ly6C^−^ and Ly6C^+^), neutrophils (CD45.2^+^ Ly6G^+^; negative for CD4, CD8, and CD19), classical monocytes (CD45.2^+^ Ly6C^high^; negative for CD4, CD8, CD19), B cells (CD45.2^+^ CD19^+^; negative for CD3 and LY6G), total T cells (CD45.2^+^ CD3^+^; negative for CD19 and LY6G), among which CD4^+^ and CD8^+^ T cells were defined (CD4^+^ CD8^-^ and CD4^-^ CD8^+^, respectively); CD4^+^ and CD8^+^ T cells were also divided in distinct activation subsets based on the expression of CD62L (CD62L^−^; CD62L^int^; CD62L^high^). The gating strategy used is represented in Supplementary Figures 1 and 2.

### Statistical Analysis

Mixed-design analysis of variance (ANOVA), the most used statistical approach in biological sciences to explore between-subject differences in repeated measures data sets, does not allow missing values. In a longitudinal experimental design, losing subjects over time (in this case due to mice death) results in extra loss of information. To overcome this gap, we took advantage of linear mixed models for repeated measures that allow exploring the progression/trajectory of the main cell populations of the peripheral immune system over time/during aging and to dissect the impact of sex on that progression. This approach prevented listwise deletion from missing data (considering that some mice died during the 18 months evaluation, as given in Supplementary Table 1). The survival rate in all sets combined was 89% for females and 44% for males, comprising an overall survival rate of 67%. All the analyses were performed with combined results from the 3 sets. Due to an atypical leukocyte profile, 4 females were excluded from the analysis (1 from set 1, 1 from set 2, and 2 from set 3) throughout the whole evaluation.

The relation between time and the percentage of cells can either be linear or quadratic. Time, represented by each month of evaluation, was introduced in the models either as a linear variable (Time) or as a quadratic variable (by calculating the time square; Time^2^). To avoid collinearity, time was centered to the mean (cTime = each timepoint – mean of time points) and only then computed cTime^2^. The cTime, sex, cTime × sex (interaction), cTime^2^, and cTime^2^ × sex (interaction) were used as fixed factors. A multivariate analysis was performed with all independent variables analyzed simultaneously. Whenever a significant interaction between Time and Sex is observed, the slope of the progression over time for females is *B*[cTime], and for males is *B*[cTime] + *B*[cTime × Sex]. Whenever *B*[cTime] presents negative values, a negative *B*[cTime^2^] indicates an acceleration and a positive *B*[cTime^2^] a deceleration over time. On the other hand, whenever *B*[cTime] presents positive values, a positive *B*[cTime^2^] indicates an acceleration and a negative *B*[cTime^2^] a deceleration over time. A random intercept per subject was introduced in the model to analyze the changes in the percentage of immune cells over time considering the within-subject correlations between the percentage of immune cells. Models were tested for various covariance structures (Level 1: repeated covariance type and Level 2: random covariance type), and the pair with the lowest Akaike’s Information Criteria was used. The estimation method used was the restricted maximum likelihood.

The variable “sets” were also included as covariates in the models (results given in Supplementary Material) to account for interexperiments variability. Additionally, the same models were tested with standardized measures, *z*-scores (computed considering the mean of each population for all the time points at each set independently). To assess the possible bias caused by missing data (mice that died before the last time point), the same analyses were performed only with survivors.

Normal distribution of the residuals from each model was checked based on skewness (≤3) and kurtosis (≤8), and all the populations present a normal distribution (excepting CD62L− populations and total NK cells).

The variance of each immune cell population at the various time points was calculated on normalized values (*z*-scores).

A one-way repeated measures ANOVA and multiple comparisons with Bonferroni correction were used to identify the differences in the expression of CD44 among the different CD4+ and CD8+ T-cell subsets (CD62L^−^; CD62L^int^; CD62L^high^).

A significant result was considered for *p* value ≤ .05. The statistical procedures were performed in IBM SPSS Version 25 (IBM Corp., Armonk, NY, USA), and the graphs were designed using Prism7 (GraphPad Software, San Diego, CA, USA).

## Results

### Age and Sex-Related Alterations in the Percentage of Blood Innate Immune Cells

**Figure 1.**
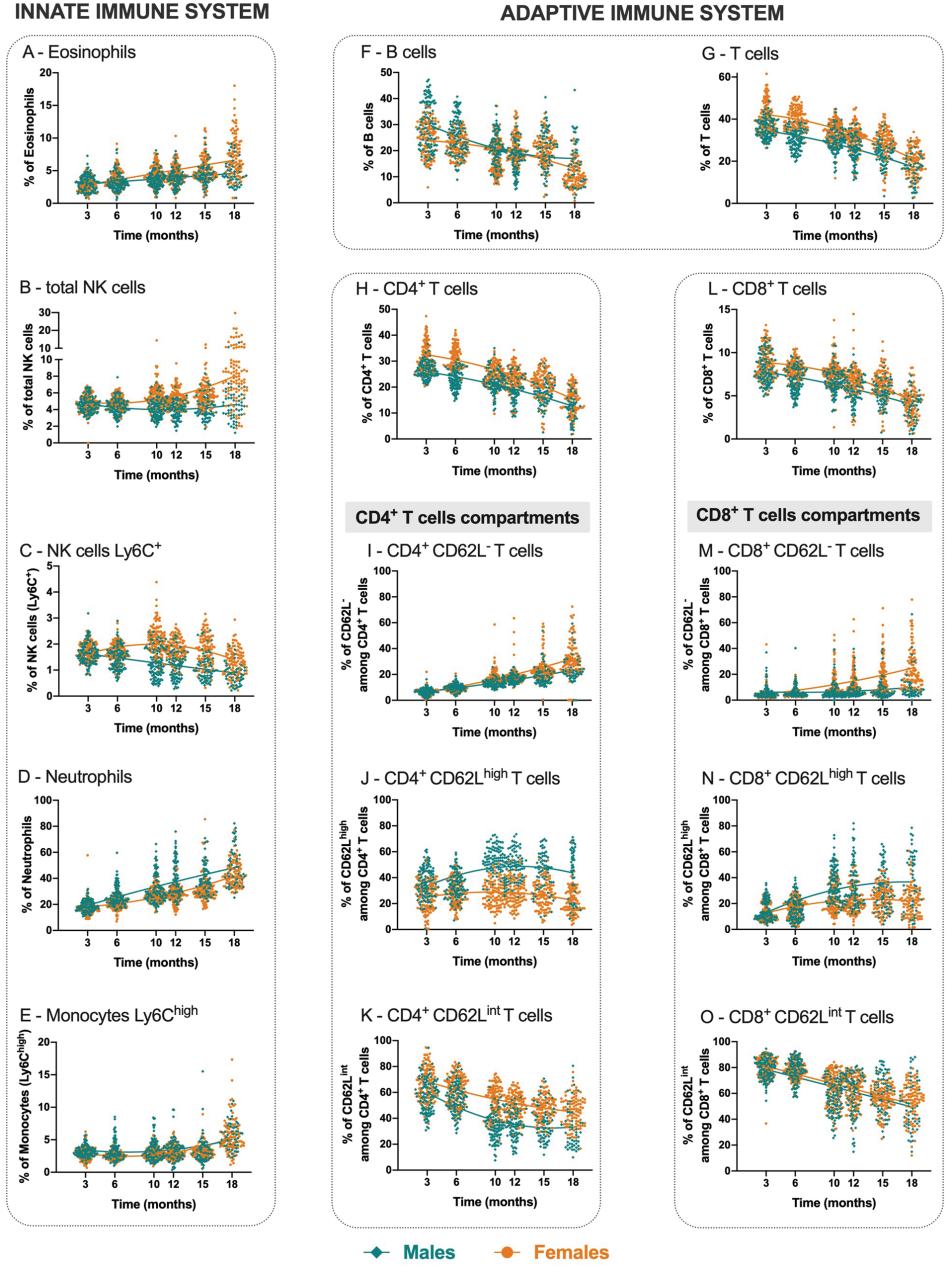
Longitudinal evaluation of blood immune cells. Percentages of the main innate (eosinophils [**A**], total NK cells [**B**], Ly6C^+^ NK cells [**C**], neutrophils [**D**], and monocytes Ly6C^high^ [**E**]) and adaptive immune cell populations over time (total B cells [**F**], total T cells [**G**], CD4^+^ T cells [**H**], CD8^+^ T cells [**L**], and activation compartments within CD4^+^ and CD8^+^ T cells: CD62L^−^ [**I** and **M**, respectively], CD62L^high^ [**J** and **N**, respectively] and CD62L^int^ [**K** and **O**, respectively]). The representation combines the results of 3 independent experimental sets. Each dot represents one animal, where males are depicted as diamonds (teal in online version) and females as circles (orange in online version). Lines represent the best-fit equation representative of the data (either linear regression or second-order polynomial [quadratic] functions).

The results are presented as a combination of 3 independent experiments (referred as set 1, set 2, and set 3). The percentage of blood innate immune cells varies with age (eosinophils, total NK cells, neutrophils, and classical monocytes increase, while Ly6C^+^ NK cells decrease). Sex dimorphism was observed in the progression of most cell populations throughout aging. Specifically:

(1) Eosinophils (Figure 1A and Table 1): males present lower cell percentages than females (*B*[sex] = −0.889, the reference category is females) and a slower linear increase over time (+0.225% per month for females vs +0.129% per month for males; *B*[cTime] = 0.225 and *B*[cTime × Sex] = −0.096).
(2) Total NK cells (Figure 1B and Table 1): males present lower cell percentages (*B*[sex] = −1.410) than females. Male percentages remained constant throughout time, while in females an increase is observed over time (+0.138% per month for females vs −0.008% for males; *B*[cTime] = 0.138 and *B*[cTime × Sex] = −0.146) with a mild acceleration (*B*[cTime^2^] = 0.007; meaning that for each month there is an increment [acceleration] of 0.007% added to *B*[cTime] for the first month, 0.028% for the second, 0.063% for the third, and so forth—same rationale for the following models). A similar profile is observed for Ly6C^−^ NK cells (Supplementary Figure 3 and Supplementary Table 2).
(3) Ly6C^+^ NK cells (Figure 1C and Table 1): males present lower cell percentages than females (*B*[sex] = −0.681) and a faster decrease over time (−0.017% per month for females vs −0.049% for males; *B*[cTime] = −0.017 and *B*[cTime × Sex] = −0.032). Females display an accelerated decrease over time, while males present only a linear decrease (−0.007% per month for females vs 0.000% for males; *B*[cTime^2^] = −0.007 and *B*[cTime^2^ × Sex] = 0.007).
(4) Neutrophils (Figure 1D and Table 1): males present higher cell percentages than females (*B*[sex] = 8.308) and a faster increase over time (+1.639% per month for females vs +1.950% for males; *B*[Time] = 1.639 and *B*[cTime × Sex] = 0.311). A slight accelerated increase in the percentage of neutrophils during aging is observed, mainly for females (+0.055% per month for females vs −0.025% for males; *B*[cTime^2^] = 0.055 and *B*[cTime^2^ × Sex] = −0.080).
(5) Monocytes Ly6C^high^ (Figure 1F and Table 1): males present higher cell percentages (*B*[sex] = 0.617), and similar increase over time is observed for both sexes (+0.176% per month; *B*[Time] = 0.176 and [cTime × Sex] not significant). The percentual increase of monocytes during aging gradually accelerates over time, more pronouncedly in females (+0.024% per month for females vs +0.015% for males; *B*[cTime^2^] = 0.024 and *B*[cTime^2^ × Sex] = −0.009).

**Table 1.** Linear Mixed Models Testing the Effect of Time (aging) and Sex on the Percentages of the Blood Main Innate Immune System Cells—a Multivariate Analysis

Dependent Variable	Parameters	Estimate (*B*)	SE	*p* †	95% CI	
					Lower Bound	Upper Bound
Eosinophils	Intercept	4.582	0.101	**<.001**	4.383	4.780
	cTime	0.225	0.017	**<.001**	0.191	0.259
	Sex*	−0.889	0.146	**<.001**	−1.177	−0.601
	cTime × Sex*	−0.096	0.028	**.001**	−0.150	−0.041
	cTime^2^	0.002	0.003	.425	−0.003	0.008
	cTime^2^ × Sex*	0.004	0.005	.331	−0.005	0.013
Total NK cells	Intercept	5.410	0.086	**<.001**	5.241	5.579
	cTime	0.138	0.016	**<.001**	0.106	0.169
	Sex*	−1.410	0.125	**<.001**	−1.655	−1.164
	cTime × Sex*	−0.146	0.025	**<.001**	−0.195	−0.097
	cTime^2^	0.007	0.003	**.012**	0.002	0.012
	cTime^2^ × Sex*	0.003	0.004	.462	−0.005	0.011
Ly6C^+^ NK cells	Intercept	1.913	0.037	**<.001**	1.839	1.986
	cTime	−0.017	0.004	**<.001**	−0.024	−0.010
	Sex*	−0.681	0.054	**<.001**	−0.787	−0.575
	cTime × Sex*	−0.032	0.006	**<.001**	−0.043	−0.020
	cTime^2^	−0.007	0.001	**<.001**	−0.008	−0.006
	cTime^2^ × Sex*	0.007	0.001	**<.001**	0.005	0.009
Neutrophils	Intercept	27.114	0.660	**<.001**	25.815	28.412
	cTime	1.639	0.075	**<.001**	1.490	1.787
	Sex*	8.308	0.951	**<.001**	6.436	10.181
	cTime × Sex*	0.311	0.123	**.012**	0.068	0.554
	cTime^2^	0.055	0.013	**<.001**	0.030	0.081
	cTime^2^ × Sex*	−0.080	0.020	**<.001**	−0.120	−0.041
Monocytes Ly6C^high^	Intercept	2.751	0.085	**<.001**	2.584	2.917
	cTime	0.176	0.066	**.008**	0.047	0.306
	Sex*	0.617	0.123	**<.001**	0.375	0.858
	cTime × Sex*	−0.060	0.093	.518	−0.243	0.123
	cTime^2^	0.024	0.001	**<.001**	0.021	0.027
	cTime^2^ × Sex*	−0.009	0.002	**<.001**	−0.014	−0.005

*Note:* CI = confidence interval; SE = standard error.

*Reference category is female.

^†^Statistically significant results (*p* < .05) are highlighted in bold.

### Age and Sex-Related Alterations in the Percentage of Blood Adaptive Immune Cells

Alike the innate immune cells, the percentage of most cell populations varies during aging in a process commonly moderated by sex.Specifically:

(1) B cells (Figure 1F and Table 2): males and females display similar cell percentages (*B*[sex] not significant), decreasing with time, more rapidly in males (−0.697% per month for females vs −0.908% for males; *B*[Time] = −0.697 and *B*[cTime × Sex] = −0.211). Interestingly, females present an accelerated decrease and males present a decelerated decrease over time (−0.035% per month for females vs +0.061% for males; *B*[cTime^2^] = −0.035 and *B*[cTime^2^ × Sex] = 0.096).
(2) T cells (Figure 1G and Table 2): males present lower cell percentages than females (*B*[sex] = −6.995) and a slower decrease over time (−1.474% per month for females vs −1.158% for males; *B*[Time] = −1.474 and *B*[cTime × Sex] = 0.316), with an overall acceleration independent of sex (*B*[cTime^2^] = −0.058 and *B*[cTime^2^ × Sex] not significant).
(3) CD4^+^ and CD8^+^ T-cell subsets (Figure 1H and L, respectively; Table 2): for both cell subsets males present lower cell percentages than females (*B*_CD4+_[Sex] = −5.403 and *B*_CD8+_[Sex] = −1.390). Moreover, while for CD8^+^ T cells males and females present a similar decrease in the percentage of cells over time (−0.296% per month; *B*[Time] = −0.296 and *B*[cTime × Sex] not significant), for CD4^+^ T cells females present a faster decrease cells than males (−1.174% per month for females vs −0.895% for males; *B*[Time] = −1.174 and *B*[cTime × Sex] = 0.279). The acceleration profile is similar in both sexes for CD4^+^ T cells (*B*[cTime^2^] = −0.037), though for CD8^+^ T cells a more pronounced acceleration is observed in females than in males (−0.018% per month for females vs −0.009% for males; *B*[cTime^2^] = −0.018 and *B*[cTime^2^ × Sex] = 0.009).

**Table 2.** Linear Mixed Models Testing the Effect of Time (aging) and Sex on the Percentages of the Blood Main Adaptive Immune System Cells—a Multivariate Analysis

Dependent Variable	Parameters	Estimate (*B*)	*SE*	*p* †	95% CI	
					Lower Bound	Upper Bound
B cells	Intercept	20.193	0.437	**<.001**	19.333	21.053
	cTime	−0.697	0.056	**<.001**	−0.806	−0.587
	Sex*	−0.230	0.635	.718	−1.477	1.018
	cTime × Sex*	−0.211	0.086	**.015**	−0.381	−0.041
	cTime^2^	−0.035	0.011	**.002**	−0.058	−0.013
	cTime^2^ × Sex*	0.096	0.017	**<.001**	0.062	0.130
T cells	Intercept	34.433	0.417	**<.001**	33.612	35.254
	cTime	−1.474	0.052	**<.001**	−1.577	−1.372
	Sex*	−6.995	0.604	**<.001**	−8.184	−5.806
	cTime × Sex*	0.316	0.082	**<.001**	0.155	0.477
	cTime^2^	−0.058	0.009	**<.001**	−0.075	−0.040
	cTime^2^ × Sex*	0.017	0.014	.221	−0.010	0.045
CD4^+^ T cells	Intercept	25.802	0.323	**<.001**	25.165	26.438
	cTime	−1.174	0.038	**<.001**	−1.250	−1.098
	Sex*	−5.403	0.469	**<.001**	−6.326	−4.480
	cTime × Sex*	0.279	0.061	**<.001**	0.159	0.399
	cTime^2^	−0.037	0.007	**<.001**	−0.050	−0.023
	cTime^2^ × Sex*	0.010	0.011	.359	−0.011	0.031
CD62L^−^ among CD4^+^ T cells	Intercept	17.174	0.402	**<.001**	16.381	17.966
	cTime	1.789	0.072	**<.001**	1.647	1.932
	Sex*	−2.682	0.581	**<.001**	−3.826	−1.538
	cTime × Sex*	−0.749	0.113	**<.001**	−0.971	−0.527
	cTime^2^	0.051	0.009	**<.001**	0.034	0.069
	cTime^2^ × Sex*	−0.051	0.014	**<.001**	−0.078	−0.023
CD62L^int^ among CD4^+^ T cells	Intercept	53.645	0.756	**<.001**	52.158	55.133
	cTime	−1.547	0.089	**<.001**	−1.723	−1.371
	Sex*	−16.141	1.115	**<.001**	−18.333	−13.949
	cTime × Sex*	−0.073	0.139	.601	−0.347	0.201
	cTime^2^	0.048	0.018	**.006**	0.014	0.083
	cTime^2^ × Sex*	0.115	0.027	**<.001**	0.062	0.168
CD62L^high^ among CD4^+^ T cells	Intercept	28.844	0.800	**<.001**	27.270	30.418
	cTime	−0.198	0.101	.051	−0.396	0.001
	Sex*	19.334	1.171	**<.001**	17.030	21.639
	cTime × Sex*	0.748	0.158	**<.001**	0.436	1.059
	cTime^2^	−0.091	0.019	**<.001**	−0.128	−0.055
	cTime^2^ × Sex*	−0.090	0.029	**.002**	−0.146	−0.033
CD8^+^ T cells	Intercept	7.480	0.149	**<.001**	7.186	7.774
	cTime	−0.296	0.067	**<.001**	−0.428	−0.164
	Sex*	−1.390	0.213	**<.001**	−1.811	−0.970
	cTime × Sex*	0.022	0.095	.816	−0.164	0.209
	cTime^2^	−0.018	0.002	**<.001**	−0.021	−0.015
	cTime^2^ × Sex*	0.009	0.003	**<.001**	0.004	0.015
CD62L^−^ among CD8^+^ T cells	Intercept	11.359	0.646	**<.001**	10.084	12.633
	cTime	1.372	0.092	**<.001**	1.189	1.554
	Sex*	−4.240	0.928	**<.001**	−6.071	−2.409
	cTime × Sex*	−1.061	0.146	**<.001**	−1.349	−0.774
	cTime^2^	0.062	0.014	**<.001**	0.034	0.089
	cTime^2^ × Sex*	−0.033	0.021	.119	−0.075	0.009
CD62L^int^ among CD8^+^ T cells	Intercept	65.384	0.898	**<.001**	63.618	67.150
	cTime	−1.950	0.103	**<.001**	−2.153	−1.746
	Sex*	−1.263	1.309	.335	−3.837	1.311
	cTime × Sex*	0.121	0.165	.464	−0.204	0.446
	cTime^2^	0.032	0.019	.092	−0.005	0.070
	cTime^2^ × Sex*	0.002	0.029	.936	−0.055	0.060
CD62L^high^ among CD8^+^ T cells	Intercept	22.334	0.932	**<.001**	20.501	24.167
	cTime	0.588	0.095	**<.001**	0.401	0.775
	Sex*	6.978	1.352	**<.001**	4.318	9.638
	cTime × Sex*	0.975	0.152	**<.001**	0.674	1.276
	cTime^2^	−0.087	0.018	**<.001**	−0.121	−0.052
	cTime^2^ × Sex*	0.024	0.027	**<.001**	−0.030	0.077

*Note:* CI = confidence interval; SE = standard error.

*Reference category is female.

^†^Statistically significant results (*p* < .05) are highlighted in bold.

CD4^+^ and CD8^+^ T cells can be divided into subsets/compartments based on their activation state. Mouse studies, based mostly on C57BL/6 mice, define T cells as: naïve, CD44^low/−^CD62L^high^; central memory, CD44^high^ CD62L^high^; and effector memory, CD44^high^ CD62L^low/−^ (19,20). However, the expression profile of CD44 and CD62L is distinct in Balb/c mice. Considering that in Balb/c mice 3 clear populations can be identified based on CD62L in CD4^+^ and CD8^+^ T cells (Supplementary Figure 2G and H), this study only used this marker to define the T-cell compartments. Within CD4^+^ T cells, CD62L^−^ cells present the highest expression of CD44, and CD62L^int^ the lowest; within CD8^+^ T cells, CD62L^−^ and CD62L^high^ present the highest expression of CD44 and CD62L^int^ the lowest (Supplementary Figure 4). The percentage of CD62L^−^ T cells increases over time (in both sexes among CD4^+^ T cells, and exclusively for females among CD8^+^ T cells); CD62L^int^ T cells decrease with aging both in CD4^+^ and CD8^+^ T cells. While females have a higher percentage of CD62L^int^ cells than males among CD4^+^ T cells; CD62L^high^ T cells increase almost exclusively on males. More precisely:

(1) CD62L^−^ T cells (Figure 1I and M; Table 2): males present lower percentages of CD62L^−^ cells among CD4^+^ and CD8^+^ T cells (*B*_CD4+CD62L_-[Sex] = −2.682 and *B*_CD8+CD62L−_[Sex] = −4.240), and these percentages increase with aging for both sexes. Interestingly, the increase of CD4^+^CD62L^−^ T cells is slower in males than in females (+1.789% per month for females vs +1.040% for males; *B*[Time] = 1.789 and *B*[cTime × Sex] = −0.749), while the increase in the CD8^+^CD62L^−^ T cells percentages is more pronouncedly observed for females (1.372% per month for females vs 0.311% for males; *B*[Time] = 1.372 and *B*[cTime × Sex] = −1.061).
(2) CD62L^high^ T cells (Figure 1J and N; Table 2): the percentage of this CD4^+^ T-cell subset is higher in males than in females (*B*[sex] = 19.334). While cell percentages decrease with aging in females, males display an increase (−0.198% per month for females vs +0.550% for males; *B*[cTime] = −0.198, *p* = .051 and *B*[cTime × Sex] = 0.748). Additionally, females present an acceleration over time, while males present a deceleration (−0.091% per month for females vs −0.181% for males; *B*[cTime^2^] = −0.091 and *B*[cTime^2^ × Sex] = −0.090). Regarding CD8^+^ T cells (Figure 1N and Table 2), males present higher percentages of CD62L^high^ cells than females (*B*[Sex] = 6.978), with a faster increase over time (0.588% per month for females vs 1.563% for males; *B*[cTime] = 0.588; *B*[cTime × Sex] = 0.975) and overall deceleration (−0.087% per month for females vs −0.063% for males; *B*[cTime^2^] = −0.087 and *B*[cTime^2^ × Sex] = +0.024).
(3) CD62L^int^ T cells (Figure 1K and O; Table 2): cell percentages decrease over time in both CD4^+^ T cells and CD8^+^ T cells (−1.547% per month [*B*_CD4+_[Time] = −1.547] and −1.959% per month [*B*_CD8+_[Time] = −1.959], respectively), with males and females presenting a similar decrease of CD62L^int^ T cells within the CD8^+^ T-cell subset (*B*[cTime × Sex] and *B*[cTime^2^ × Sex] not significant), and males presenting a more pronounced deceleration of CD62L^int^ T cells within the CD4^+^ T-cell subsets overtime than females (0.046% per month for females vs 0.161% for males; *B*[cTime^2^] = +0.046, *B*[cTime^2^ × Sex] = +0.115, *B*[cTime × Sex] not significant). Males present lower percentages of CD62L^int^ cells within the CD4^+^ T-cell subsets than females (*B*[Sex] = −16 141) and similar profiles regarding the percentages of CD62L^int^ CD8^+^ T cells (*B*[Sex] not significant).

### Control of the Interexperimental Set Variability and Exclusion of Mice That Died During the Course of the Experiment Have No Impact on the Overall Observations

Because we compiled the data from 3 independent experiments, and to control for the heterogeneity, the percentages of cells from each of the 3 sets were standardized by calculating the *z*-scores. As shown in Supplementary Figure 5, the progression for standardized measures is similar to that presented in Figure 1 (unstandardized measures). The results from the linear mixed models are identical in the standardized and unstandardized measures except for (a) B cells: sex becomes a significant parameter and the significance in the interaction between time and sex is lost when considering standardized values; and (b) CD4^+^CD62L^int^ T cells: the quadratic effect of time is lost when considered standardized values (data not shown).

To control for a potential effect of individual experimental sets in the linear mixed models with unstandardized values, the variable “set” was also included (Supplementary Tables 3 and 4). When controlling for the “set” effect, the significant parameters remain the same with estimates within the 95% confidence interval of the first model.

**Figure 2.**
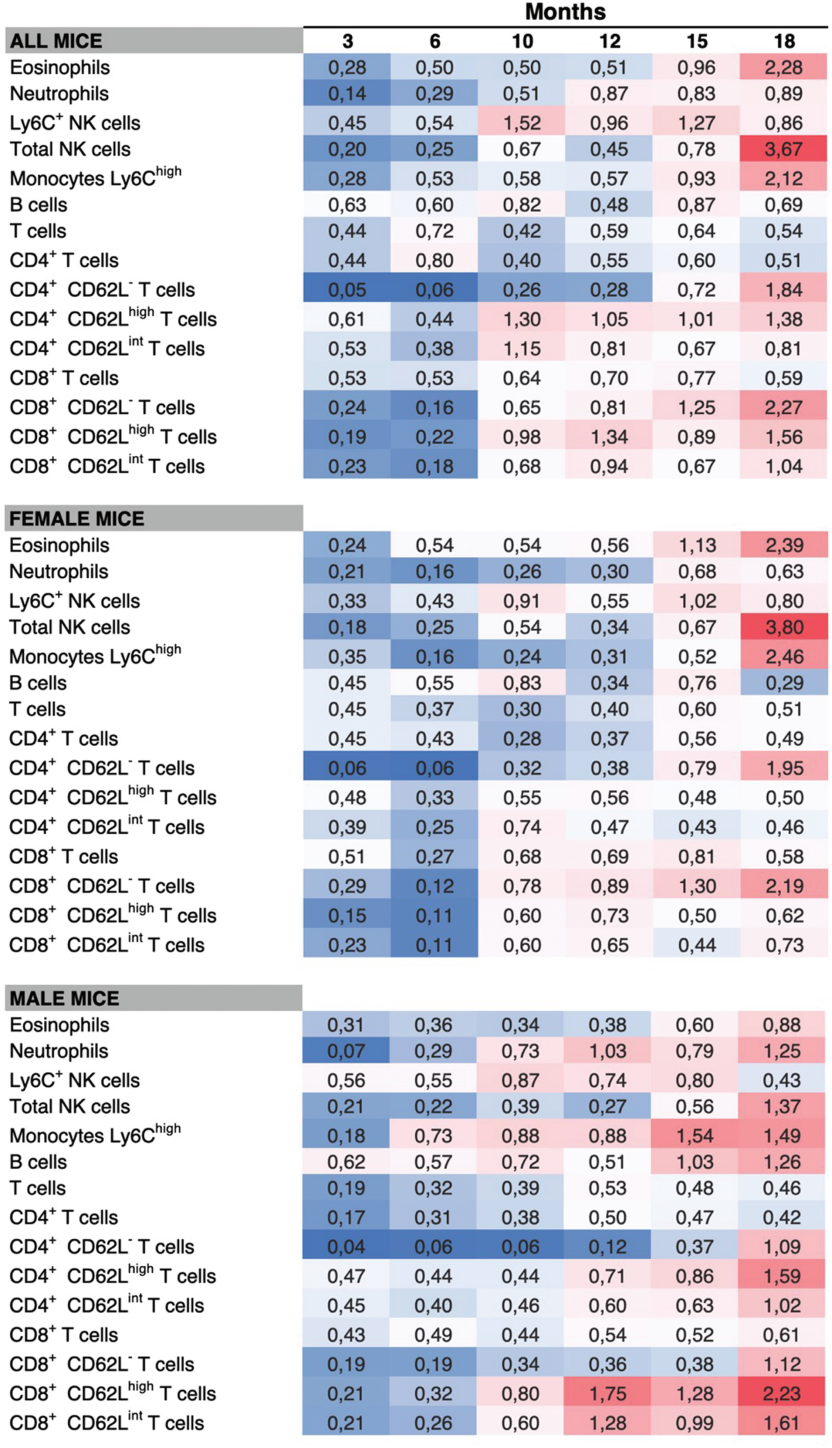
Variance of each immune cell population at various time points (overall—top panel; only on females—middle panel; only on males—bottom panel). Variance calculated on normalized values (*z*-scores). Dark grey corresponds to the lowest and highest variances (in online version blue corresponds to the lowest variances and red to the highest).

Moreover, the total variability within immune cell populations increases with aging, as demonstrated by the increased variance in most populations at 18 months of age (Figure 2). Total NK cells present the highest variability observed at 18 months of age, more evidently denoted in female mice. Exploring the covariates associated with this age-related increased variability will contribute to the understanding of intervariability in the aging process.

As the survival rate, especially in males, is relatively low (44%), it is conceivable that mice that died during the experiments might be different and not representative of the overall population. To explore the possible impact of a listwise exclusion of all mice that died throughout the experiment, linear mixed models were also built using only the survivors; no impact was observed on the main conclusions (Supplementary Figure 6).

## Discussion

This longitudinal study shows that the percentages of immune cells in mice vary with aging and that males and females show distinct progression profiles. More specifically, the percentage of neutrophils, monocytes, eosinophils, and total NK cells increases with age, while that of Ly6C^+^ NK cells, B and T cells (both CD4^+^ and CD8^+^) decreases. These observations reveal an overall decrease of adaptive and an increase of innate immune cells, a signature that is in line with some of the known age-related changes of the immune system that occur in humans (2,12,17,21–24). Of relevance, a skew in hematopoiesis during aging, favoring the myeloid lineage, has been shown both in humans and rodents (25,26), which can contribute to the observed increased proportion of circulating innate cells. In the same line of evidence, both aged humans and mice present increased levels of total monocytes in comparison to young subjects (2,17). However, with respect to classical monocytes, the population explored in this study, humans and inbred mice seem to differ: in humans, CD14^high^ decrease or remain constant with aging (11,12), whereas in mice, Ly6C^high^ are increased in old mice, as demonstrated here and by others (17). This discrepancy might be related to genetic variability since CD-1 mice, which present higher genetic variability than inbred strains, also display decreased numbers of classical monocytes in aged mice (27). Moreover, a reduction in monocytes’ chemotaxis has been described (3), which might explain the observed accumulation of these cells in circulation. The percentage of human neutrophils increases with age (21), similarly to what we observed here for mice. Neutrophils’ capacity to migrate to inflamed tissues is also impaired in aged animals (28,29), potentially contributing to their accumulation in the blood. Concerning NK cells, we observed an overall decrease of Ly6C^+^ NK cells and an increase of total NK cells. In humans, an increase in total NK cells during aging has been extensively reported (12,30–32) as we show for female mice. This is in contrast with reports from experiments using C57BL/6 mice in which NK cells are selected as NK1.1^+^ cells (33), a molecule not expressed in Balb/c mice (34). We, and others using Balb/c mice (35), define NK cells as CD49b^+^ (and negative for CD4, CD8, CD19, and Ly6G). Recently, the expression of CD49b was seen to occur also in atypical innate lymphoid cells (ILC1) (36), which might indicate that we are selecting a broader population of cells rendering the comparison between studies difficult. An NK cell subset based on the expression of Ly6C was identified, even though the expression of this marker on NK cells is still poorly characterized. It was reported that mature NK cells (NK1.1^+^ CD11b^+^) expressing Ly6C present a resting/inert phenotype with a poor proliferative potential (37), and that memory NK cells express high levels of Ly6C (and low levels of CD27) upon viral infection (38,39). Curiously, CD27^−^ CD11b^+^ NK cells, considered the most mature cells, were shown to decrease in the blood of aged mice (15), which is in line with our findings. However, the mouse CD27^−^ NK cells present some similarities with human CD56^dim^ NK cells (40), which have been described to be increased in older individuals (32,41). Considering the absence of CD56 expression in mice (40), additional markers should be used to better characterize the dynamics of the various NK cells subpopulations during aging to allow a more accurate comparison with humans.

As for the adaptive immune system, it has been extensively described that human B and T (both CD4^+^ and CD8^+^ subsets) percentages and absolute numbers decrease with aging (11,12,22,23), as observed here in mice. Moreover, studies in rats and mice also evidenced a decrease in splenic T cells (or CD4^+^ T cells) in old animals (16,42).

Considering the importance to explore sex as a biological variable that might influence preclinical research (43) and the lack of reports tackling this issue in mouse healthy aging, we explored sex-related differences in the percentage of immune cells over time.

We show that sex is a significant and confounding factor in the progression of the main blood populations of the immune system evaluated. Males present overall higher percentages of neutrophils and classical monocytes compared to females. By contrast, females present higher percentages of total T cells, and both CD4^+^ and CD8^+^ T cells, eosinophils, and NK cells. Even though males and females present similar percentages of B cells, their progression over time reveals opposing acceleration profiles. Altogether, this clearly demonstrates an age-related sexual dimorphism in the proportion of immune cells in circulation. Sex differences in basal conditions, as shown here, are still poorly characterized, though extensively explored during immune responses both in humans and in animal models. In studies comparing the immune response in males versus females, females usually present a stronger immune response resulting in a more efficient pathogen clearance but increased susceptibility to autoimmunity (9,10). Additionally, in aged individuals, women have higher genomic accessibility for genes (ATAC-seq) related to adaptive immunity and lower for monocytes and inflammation-related genes than men (11). As described here, the percentages of total T cells, both CD4^+^ and CD8^+^ subsets, are lower in males and decrease during aging in both sexes. Accordingly, human cross-sectional studies demonstrated an identical profile, even though the impact of sex was only observed for total T cells and CD4^+^ T cells (11,12). These findings might be related to the fact that thymic function decreases with aging differently in men and women. As no direct method is available to quantify thymic function, this has been estimated using various surrogates, of which the quantification of T-cell receptor excision circles (TRECs) in peripheral blood (44) is the most used. The decrease of TRECs with age, from 20 to 70 years of age, is greater in men than in women (31), thus suggesting an accelerated decrease of thymic function in men, which might explain the greater percentages of T cells in women.

The most well-described age-related alteration in the adaptive immune system, mainly in humans, is the shift in the T-cell compartments from a naïve into memory and effector phenotypes (5,6,45). For strains comparisons, a note is necessary on the classification of T-cell compartments in mice. The classical classification of T-cell compartments in mice, retrieved essentially from the C57BL/6 strain, is based on the expression of CD44 and CD62L (naïve T cells as CD44^low/−^CD62L^high^, central memory T cells as CD44^high^ CD62L^high^, and effector memory T cells as CD44^high^ CD62L^low/−^) (19,20). However, the expression profile of CD44 and CD62L is distinct in Balb/c mice, as shown by others (46) and also here. Balb/c mice present higher expression of CD62L than C57BL/6 mice (46), possibly explaining some of the strain differences. In Balb/c mice, 3 distinct populations based on the expression of CD62L are defined: CD62L^−^, CD62L^int^, and CD62L^high^. Considering the median fluorescence intensity of CD44 on each population, we propose that CD62L^−^ T cells are effector memory cells due to their highest expression of CD44; CD62L^high^ T cells are central memory cells due to their intermediate expression of CD44; and CD62L^int^ T cells are naïve cells due to their lowest expression of CD44 (19,20). Using this classification, we observe that CD62L^−^ T cells (effector memory cells) increase over time (in both sexes among CD4^+^ T cells, and exclusively for females on CD8^+^ T cells); CD62L^int^ T cells (naïve cells) decrease with aging both in CD4^+^ and CD8^+^ T cells, where females have clearly more naïve CD4^+^ T cells; and CD62L^high^ T cells (central memory cells) increase almost exclusively on males. Concordantly, in humans, the decrease in naïve T cells is more pronounced in CD8^+^ T cells, with women displaying higher relative amounts of naïve T cells than men (11,12,47). On the other hand, men present a higher proportion of effector T cells than women (47), mostly at young ages (11). To note, here we present the proportion of cells among either CD4^+^ or CD8^+^ T cells, while human studies present mostly absolute counts or percentages among total peripheral blood mononuclear cells (11,47), which can explain some of the inconsistencies found. Moreover, the dynamics of T-cell compartments seem to display considerable species differences, which is of relevance when age is considered. For instance, the proportion of T-cell compartments in the adult mouse is more similar to that of neonate humans than adults (48). Overall, irrespectively from the T-cell compartments analyzed, the alterations in the proportion of cells are strongly influenced by sex, reinforcing the need to investigate both males and females when addressing the impact of aging on T cells.

Potential mechanisms responsible for sex-related differences are likely related to the effects of sex hormones on the immune system and to genetic factors, as described elsewhere (8,9,49). The increased variability in the proportion of immune cells with aging, which has also been reported at the transcriptional level (50), reinforces the need to consider individual progressions when analyzing biological parameters in age-related research. Longitudinal studies and mixed-effects models are 2 strategies that should be implemented more often when studying immune system aging.

The concordance across human and mouse studies suggests that some of the aging and sex-specific signatures described are conserved across species, which gives the confidence to use animal models when seeking further understanding of the sex-related discrepancies in immune system aging.

Taken together, this longitudinal study comprehensively explores the evolution of the main immune cell populations during aging, revealing an overall decrease in the adaptive immune cells and an increase in innate cells. It also reveals that sex plays an essential role in the mouse immune system aging, as described in humans, and must be taken into account when designing and planning experiments with rodents.

## Supplementary Material

glab330_suppl_Supplementary_Material

## Data Availability

The data that support the findings of this study are available from the corresponding author upon reasonable request.

## References

[1] Shaw AC, Goldstein DR, Montgomery RR. Age-dependent dysregulation of innate immunity. Nat Rev Immunol. 2013;13(12):875–887. doi:10.1038/nri3547 24157572 PMC4096436

[2] Della Bella S, Bierti L, Presicce P, et al. Peripheral blood dendritic cells and monocytes are differently regulated in the elderly. Clin Immunol. 2007;122(2):220–228. doi:10.1016/j.clim.2006.09.012 17101294

[3] Panda A, Arjona A, Sapey E, et al. Human innate immunosenescence: causes and consequences for immunity in old age. Trends Immunol. 2009;30(7):325–333. doi:10.1016/j.it.2009.05.004 19541535 PMC4067971

[4] Mocchegiani E, Giacconi R, Cipriano C, Malavolta M. NK and NKT cells in aging and longevity: role of zinc and metallothioneins. J Clin Immunol. 2009;29(4):416–425. doi:10.1007/s10875-009-9298-4 19408107

[5] Saule P, Trauet J, Dutriez V, Lekeux V, Dessaint JP, Labalette M. Accumulation of memory T cells from childhood to old age: central and effector memory cells in CD4(+) versus effector memory and terminally differentiated memory cells in CD8(+) compartment. Mech Ageing Dev. 2006;127(3):274–281. doi:10.1016/j.mad.2005.11.001 16352331

[6] Nikolich-Žugich J . Aging of the T cell compartment in mice and humans: from no naive expectations to foggy memories. J Immunol. 2014;193(6):2622–2629. doi:10.4049/jimmunol.1401174 25193936 PMC4157314

[7] Britanova OV, Putintseva EV, Shugay M, et al. Age-related decrease in TCR repertoire diversity measured with deep and normalized sequence profiling. J Immunol. 2014;192(6):2689–2698. doi:10.4049/jimmunol.1302064 24510963

[8] Gubbels Bupp MR, Potluri T, Fink AL, Klein SL. The confluence of sex hormones and aging on immunity. Front Immunol. 2018;9:1269. doi:10.3389/fimmu.2018.01269 29915601 PMC5994698

[9] Klein SL, Flanagan KL. Sex differences in immune responses. Nat Rev Immunol. 2016;16(10):626–638. doi:10.1038/nri.2016.90 27546235

[10] Giefing-Kröll C, Berger P, Lepperdinger G, Grubeck-Loebenstein B. How sex and age affect immune responses, susceptibility to infections, and response to vaccination. Aging Cell. 2015;14(3):309–321. doi:10.1111/acel.12326 25720438 PMC4406660

[11] Márquez EJ, Chung CH, Marches R, et al. Sexual-dimorphism in human immune system aging. Nat Commun. 2020;11(1):751. doi:10.1038/s41467-020-14396-9 32029736 PMC7005316

[12] Patin E, Hasan M, Bergstedt J, et al.; Milieu Intérieur Consortium. Natural variation in the parameters of innate immune cells is preferentially driven by genetic factors. Nat Immunol. 2018;19(3):302–314. doi:10.1038/s41590-018-0049-7 29476184

[13] Strindhall J, Skog M, Ernerudh J, et al. The inverted CD4/CD8 ratio and associated parameters in 66-year-old individuals: the Swedish HEXA immune study. Age (Dordr). 2013;35(3):985–991. doi:10.1007/s11357-012-9400-3 22415616 PMC3636392

[14] Abdullah M, Chai PS, Chong MY, et al. Gender effect on in vitro lymphocyte subset levels of healthy individuals. Cell Immunol. 2012;272(2):214–219. doi:10.1016/j.cellimm.2011.10.009 22078320

[15] Nair S, Fang M, Sigal LJ. The natural killer cell dysfunction of aged mice is due to the bone marrow stroma and is not restored by IL-15/IL-15Rα treatment. Aging Cell. 2015;14(2):180–190. doi:10.1111/acel.12291 25399821 PMC4364830

[16] El-Naseery NI, Mousa HSE, Noreldin AE, El-Far AH, Elewa YHA. Aging-associated immunosenescence via alterations in splenic immune cell populations in rat. Life Sci. 2020;241:117168. doi:10.1016/j.lfs.2019.117168 31838133

[17] Puchta A, Naidoo A, Verschoor CP, et al. TNF drives monocyte dysfunction with age and results in impaired anti-pneumococcal immunity. PLoS Pathog. 2016;12(1):e1005368. doi:10.1371/journal.ppat.1005368 26766566 PMC4713203

[18] Faul F, Erdfelder E, Lang AG, Buchner A. G*Power 3: a flexible statistical power analysis program for the social, behavioral, and biomedical sciences. Behav Res Methods. 2007;39(2):175–191. doi:10.3758/bf03193146 17695343

[19] Rosenblum MD, Way SS, Abbas AK. Regulatory T cell memory. Nat Rev Immunol. 2016;16(2):90–101. doi:10.1038/nri.2015.1 26688349 PMC5113825

[20] Del Zotto G, Principi E, Antonini F, et al Comprehensive phenotyping of peripheral blood T lymphocytes in healthy mice. Cytometry A. 2021;99(3): 243–250. doi:10.1002/cyto.a.24246 33098601

[21] Valiathan R, Ashman M, Asthana D. Effects of ageing on the immune system: infants to elderly. Scand J Immunol. 2016;83(4):255–266. doi:10.1111/sji.12413 26808160

[22] Paganelli R, Quinti I, Fagiolo U, et al. Changes in circulating B cells and immunoglobulin classes and subclasses in a healthy aged population. Clin Exp Immunol. 1992;90(2):351–354. doi:10.1111/j.1365-2249.1992.tb07954.x 1424294 PMC1554614

[23] Muggen AF, de Jong M, Wolvers-Tettero ILM, et al. The presence of CLL-associated stereotypic B cell receptors in the normal BCR repertoire from healthy individuals increases with age. Immun Ageing. 2019;16:22. doi:10.1186/s12979-019-0163-x 31485252 PMC6714092

[24] Chong Y, Ikematsu H, Yamaji K, et al. CD27(+) (memory) B cell decrease and apoptosis-resistant CD27(−) (naive) B cell increase in aged humans: implications for age-related peripheral B cell developmental disturbances. Int Immunol. 2005;17(4):383–390. doi:10.1093/intimm/dxh218 15724062

[25] Cho RH, Sieburg HB, Muller-Sieburg CE. A new mechanism for the aging of hematopoietic stem cells: aging changes the clonal composition of the stem cell compartment but not individual stem cells. Blood. 2008;111(12):5553–5561. doi:10.1182/blood-2007-11-123547 18413859 PMC2424153

[26] Rundberg Nilsson A, Soneji S, Adolfsson S, Bryder D, Pronk CJ. Human and murine hematopoietic stem cell aging is associated with functional impairments and intrinsic megakaryocytic/erythroid bias. PLoS One. 2016;11(7):e0158369. doi:10.1371/journal.pone.0158369 27368054 PMC4930192

[27] Strohacker K, Breslin WL, Carpenter KC, McFarlin BK. Aged mice have increased inflammatory monocyte concentration and altered expression of cell-surface functional receptors. J Biosci. 2012;37(1):55–62. doi:10.1007/s12038-011-9169-z 22357203

[28] Wenisch C, Patruta S, Daxböck F, Krause R, Hörl W. Effect of age on human neutrophil function. J Leukoc Biol. 2000;67(1):40–45. doi:10.1002/jlb.67.1.40 10647996

[29] Brubaker AL, Rendon JL, Ramirez L, Choudhry MA, Kovacs EJ. Reduced neutrophil chemotaxis and infiltration contributes to delayed resolution of cutaneous wound infection with advanced age. J Immunol. 2013;190(4):1746–1757. doi:10.4049/jimmunol.1201213 23319733 PMC3563860

[30] Schindowski K, Fröhlich L, Maurer K, Müller WE, Eckert A. Age-related impairment of human T lymphocytes’ activation: specific differences between CD4(+) and CD8(+) subsets. Mech Ageing Dev. 2002;123(4):375–390. doi:10.1016/s0047-6374(01)00396-7 11744048

[31] Clave E, Araujo IL, Alanio C, et al Human thymopoiesis is influenced by a common genetic variant within the TCRA-TCRD locus. Sci Transl Med. 2018;10(457):eaao2966. doi:10.1126/scitranslmed.aao2966 30185651

[32] Almeida-Oliveira A, Smith-Carvalho M, Porto LC, et al. Age-related changes in natural killer cell receptors from childhood through old age. Hum Immunol. 2011;72(4):319–329. doi:10.1016/j.humimm.2011.01.009 21262312

[33] Beli E, Duriancik DM, Clinthorne JF, Lee T, Kim S, Gardner EM. Natural killer cell development and maturation in aged mice. Mech Ageing Dev. 2014;135:33–40. doi:10.1016/j.mad.2013.11.007 24361677 PMC4043280

[34] Carlyle JR, Mesci A, Ljutic B, et al. Molecular and genetic basis for strain-dependent NK1.1 alloreactivity of mouse NK cells. J Immunol. 2006;176(12):7511–7524. doi:10.4049/jimmunol.176.12.7511 16751398

[35] Arase H, Saito T, Phillips JH, Lanier LL. Cutting edge: the mouse NK cell-associated antigen recognized by DX5 monoclonal antibody is CD49b (alpha 2 integrin, very late antigen-2). J Immunol. 2001;167(3):1141–1144. doi:10.4049/jimmunol.167.3.1141 11466327

[36] Wong E, Xu RH, Rubio D, et al. Migratory dendritic cells, group 1 innate lymphoid cells, and inflammatory monocytes collaborate to recruit NK cells to the virus-infected lymph node. Cell Rep. 2018;24(1):142–154. doi:10.1016/j.celrep.2018.06.004 29972776 PMC6232077

[37] Omi A, Enomoto Y, Kiniwa T, Miyata N, Miyajima A. Mature resting Ly6C(high) natural killer cells can be reactivated by IL-15. Eur J Immunol. 2014;44(9):2638–2647. doi:10.1002/eji.201444570 24995967

[38] Cerwenka A, Lanier LL. Natural killer cell memory in infection, inflammation and cancer. Nat Rev Immunol. 2016;16(2):112–123. doi:10.1038/nri.2015.9 26806484

[39] Sun JC, Beilke JN, Lanier LL. Adaptive immune features of natural killer cells. Nature. 2009;457(7229):557–561. doi:10.1038/nature07665 19136945 PMC2674434

[40] Hayakawa Y, Huntington ND, Nutt SL, Smyth MJ. Functional subsets of mouse natural killer cells. Immunol Rev. 2006;214:47–55. doi:10.1111/j.1600-065X.2006.00454.x 17100875

[41] Hazeldine J, Hampson P, Lord JM. Reduced release and binding of perforin at the immunological synapse underlies the age-related decline in natural killer cell cytotoxicity. Aging Cell. 2012;11(5):751–759. doi:10.1111/j.1474-9726.2012.00839.x 22642232

[42] Xie J, Zhang J, Wu H, et al. The influences of age on T lymphocyte subsets in C57BL/6 mice. Saudi J Biol Sci. 2017;24(1):108–113. doi:10.1016/j.sjbs.2016.09.002 28053579 PMC5198989

[43] Miller LR, Marks C, Becker JB, et al. Considering sex as a biological variable in preclinical research. FASEB J. 2017;31(1):29–34. doi:10.1096/fj.201600781R 27682203 PMC6191005

[44] Dion ML, Sékaly RP, Cheynier R. Estimating thymic function through quantification of T-cell receptor excision circles. Methods Mol Biol. 2007;380:197–213. doi:10.1007/978-1-59745-395-0_12 17876095

[45] Goronzy JJ, Weyand CM. T cell development and receptor diversity during aging. Curr Opin Immunol. 2005;17(5):468–475. doi:10.1016/j.coi.2005.07.020 16098723

[46] Tu L, Poe JC, Kadono T, et al. A functional role for circulating mouse L-selectin in regulating leukocyte/endothelial cell interactions in vivo. J Immunol. 2002;169(4):2034–2043. doi:10.4049/jimmunol.169.4.2034 12165530

[47] Yan J, Greer JM, Hull R, et al. The effect of ageing on human lymphocyte subsets: comparison of males and females. Immun Ageing. 2010;7:4. doi:10.1186/1742-4933-7-4 20233447 PMC2858100

[48] Beura LK, Hamilton SE, Bi K, et al. Normalizing the environment recapitulates adult human immune traits in laboratory mice. Nature. 2016;532(7600):512–516. doi:10.1038/nature17655 27096360 PMC4871315

[49] Gubbels Bupp MR . Sex, the aging immune system, and chronic disease. Cell Immunol. 2015;294(2):102–110. doi:10.1016/j.cellimm.2015.02.002 25700766

[50] Martinez-Jimenez CP, Eling N, Chen HC, et al. Aging increases cell-to-cell transcriptional variability upon immune stimulation. Science. 2017;355(6332):1433–1436. doi:10.1126/science.aah4115 28360329 PMC5405862

