# Supplementary material for: Age-Related Sexual Dimorphism on the Longitudinal Progression of Blood Immune Cells in BALB/cByJ Mice
Source: J Gerontol A Biol Sci Med Sci. 2021 Nov 6;77(5):883–91. doi: 10.1093/gerona/glab330 (PMC9071472; doi:10.1093/gerona/glab330)
Supplement: glab330_suppl_Supplementary_Material [file glab330_suppl_supplementary_material.pdf]

Age-related sexual dimorphism on the longitudinal progression of blood immune cells in BALB/cByJ mice

Cláudia Serre-Miranda, PhD [a,b], Susana Roque, PhD [a,b], Palmira Barreira-Silva, PhD [a,b], Claudia Nobrega, PhD [a,b], Neide Vieira, PhD [a,b], Patrício Costa, PhD [a,b], Joana Almeida Palha, PhD [a,b], Margarida Correia-Neves, PhD [a,b,*]

[a] Life and Health Sciences Research Institute (ICVS), School of Medicine, University of Minho, Braga, Portugal

[b] ICVS/3B's - PT Government Associate Laboratory, Braga/Guimarães, Portugal

***Corresponding author:** Margarida Correia-Neves, Life and Health Sciences Research Institute, School of Medicine, University of Minho, Campus de Gualtar, 4710-057 Braga, Portugal.

E-mail: mcorreianeves@med.uminho.pt; telephone number: +351 253 604 807.

SUPPLEMENTARY INFORMATION

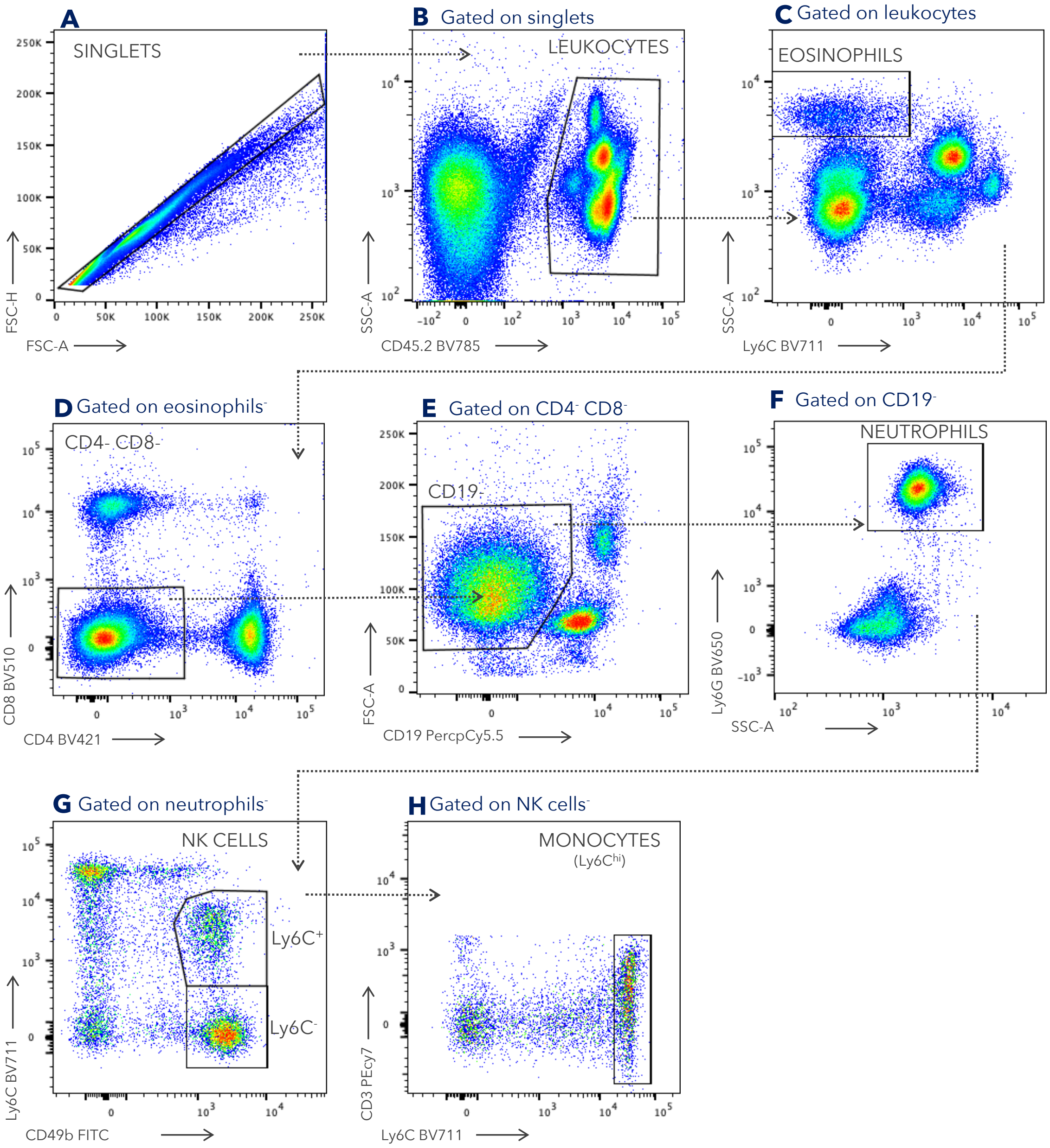

Supplementary Figure 1 Flow cytometry gating strategy of the blood main innate immune cells. The analysis initiated by selecting singlets (A). Definition of cell types was as follows: leukocytes as CD45.2^{+} cells (B); eosinophils as Ly6C^{-} and SSC^{high} (C); upon excluding eosinophils and selecting CD4^{-}CD8^{-} (D) and CD19^{-} (E), neutrophils were defined as Ly6G^{+} cells (F); among neutrophils, NK cells were subdivided as CD49b+Ly6C+ and CD49b+Ly6C-(G); after excluding all the aforementioned cells, activated monocytes were defined as Ly6Chigh cells (H). The definition of a positive or negative population for each marker was based on their expression density; the gating strategy was standardized between the 3 sets and the different timepoints.

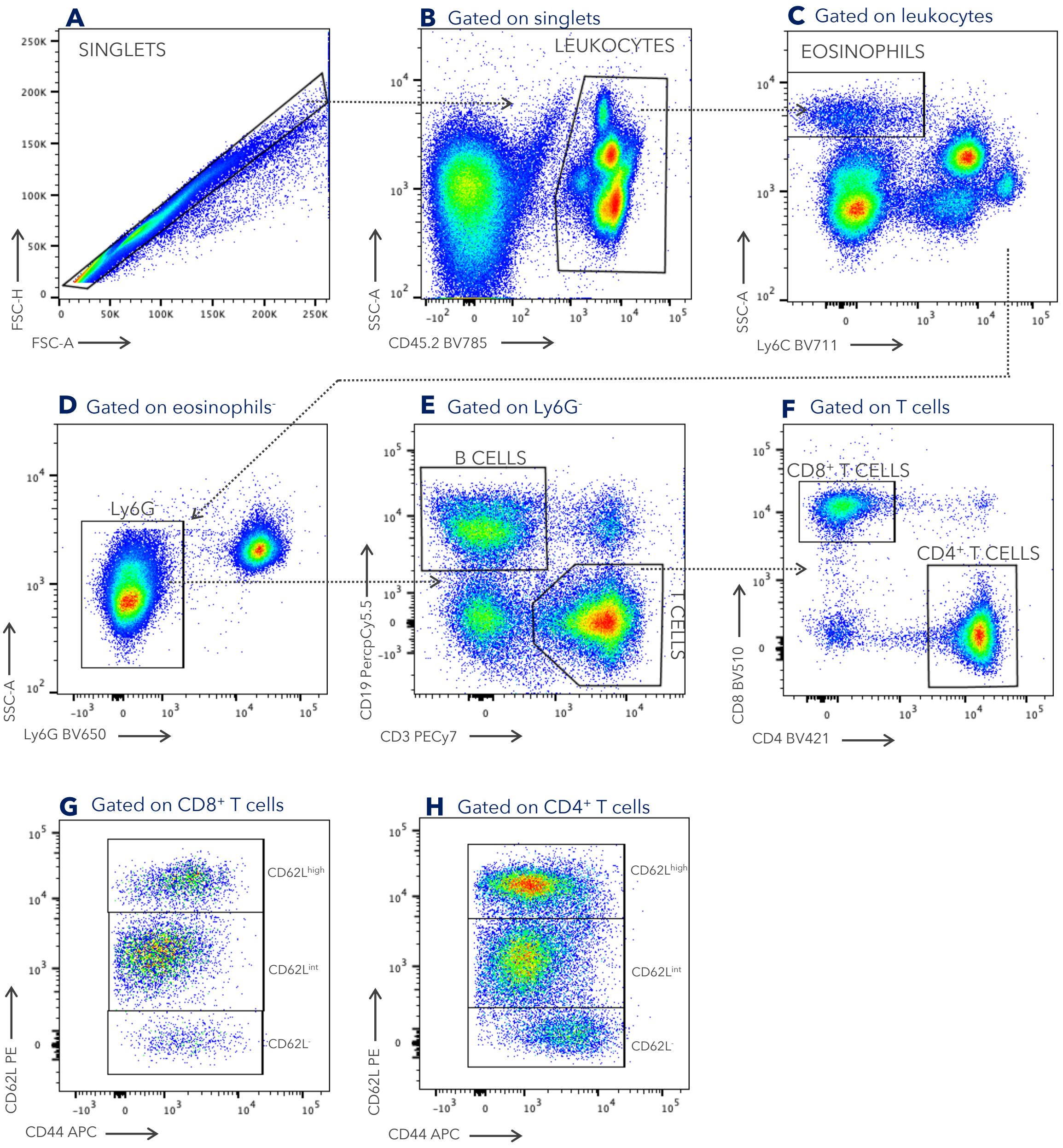

Supplementary Figure 2 Flow cytometry gating strategy of the blood main adaptive immune cells. The analysis started by selecting singlets (A). Definition of cell types was as follows: leukocytes as CD45.2$^+$ cells (B); after excluding eosinophils (Ly6C$^-$SSC^{high}) (C) and selecting Ly6G$^-$ cells (D), B cells were defined as CD19$^+$CD3$^-$ and T cells as CD19$^-$CD3$^+$ (E); among T cells, CD4$^+$ T cells were defined as CD4$^+$CD8$^-$ and CD8$^+$ T cells as CD4$^-$CD8$^+$ (F); the different activation state/cellular compartments among either CD8$^+$ T cells or CD4$^+$ T cells were defined based on the expression of CD62L (G and H, respectively). The definition of a positive or negative population for each marker was based on their expression density; the gating strategy was standardized between the 3 sets and the different timepoints.

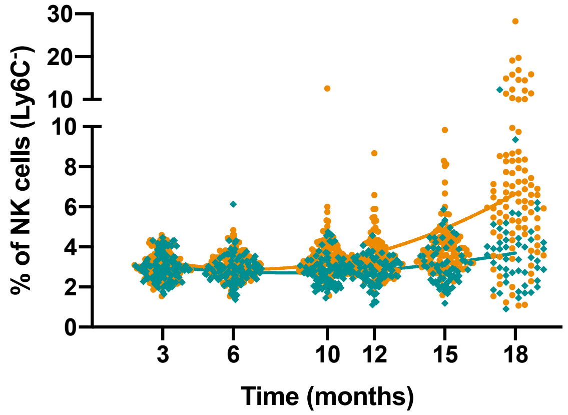

Supplementary Figure 3. Longitudinal evaluation of blood Ly6C- NK cells. The representation combines the results from the 3 experimental sets. Each dot represents an animal, where males are depicted as teal diamonds and females are depicted as orange circles. Lines represent the best fit equation representative of the data [either linear regression or second order polynomial (quadratic) functions].

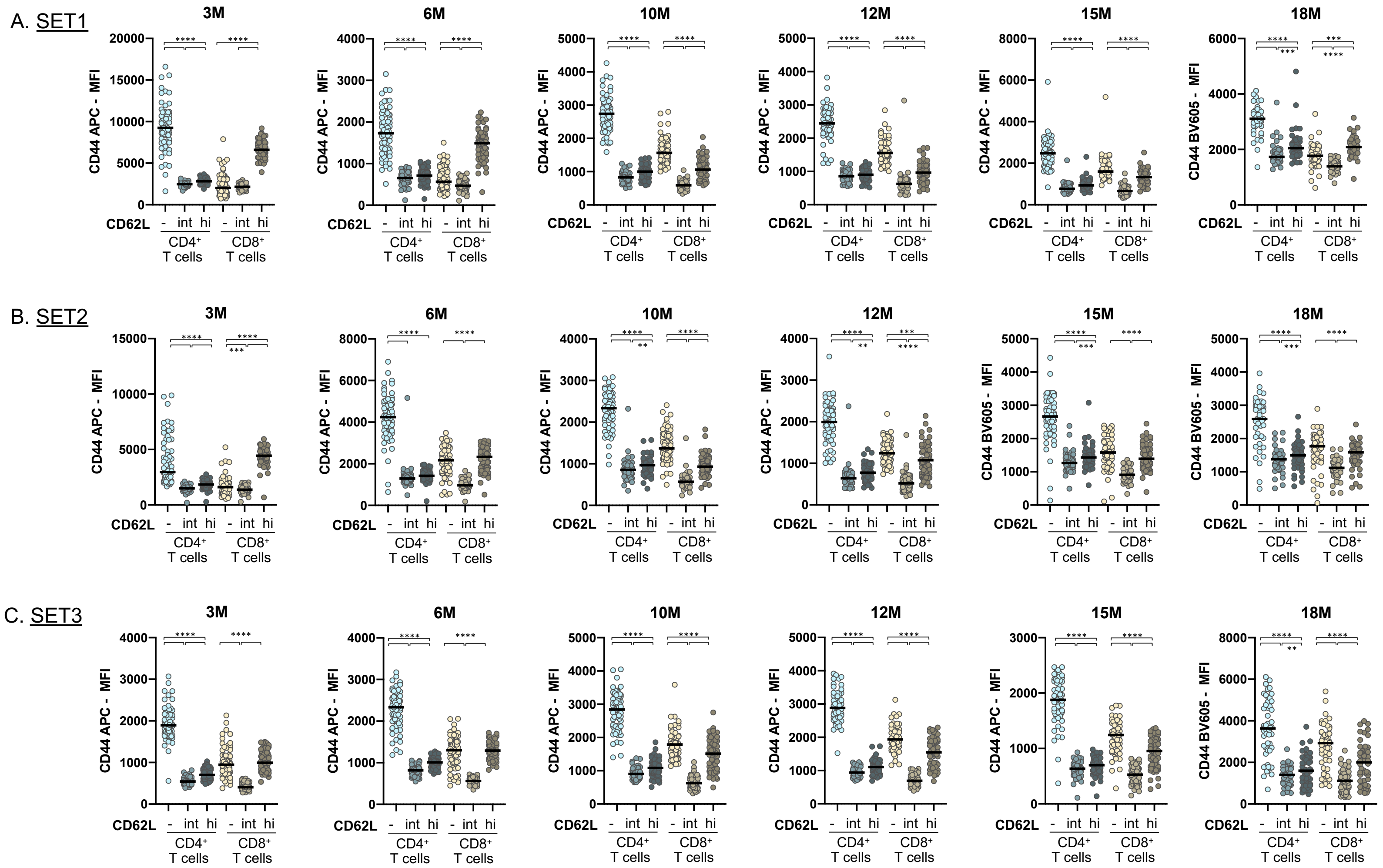

Supplementary Figure 4. Median fluorescence intensity (MFI) of CD44 APC (or BV605) for the various CD4^{+} and CD8^{+} T cell subsets based on the expression of CD62L. Representation of MFI values for CD44 at each timepoint for each experimental set independently – set1 (A), set2 (B), set3 (C). Each dot represents an animal and the lines the average of the group. One-way repeated measures ANOVA and multiple comparisons with Bonferroni correction were used to identify the differences on the expression of CD44 among the different CD4^{+} and CD8^{+} T cell subsets (CD62L^{-}; CD62L^{int}; CD62L^{high}): **p<0.01; ***p<0.001; ****p<0.0001.

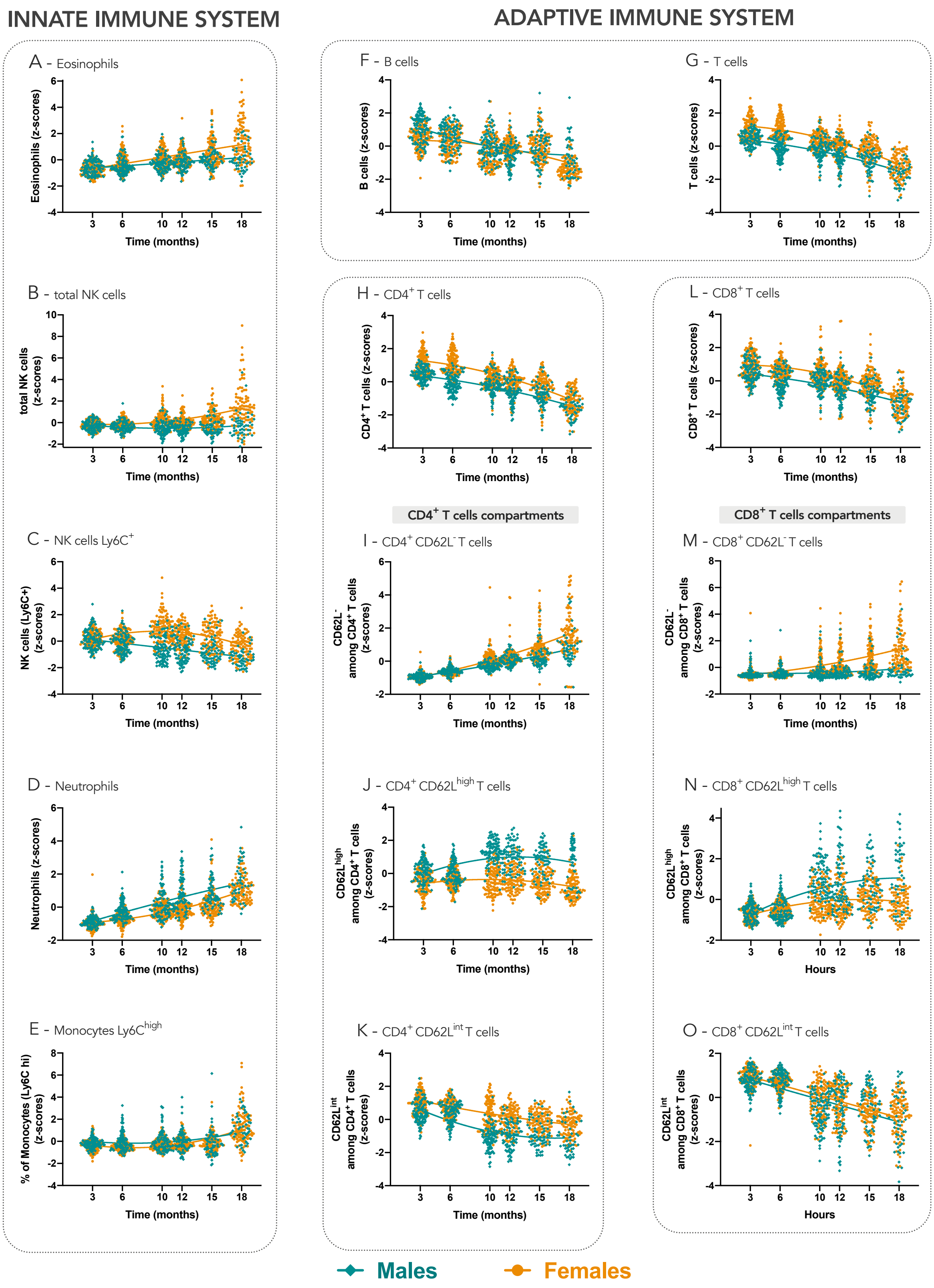

Supplementary Figure 5. Longitudinal evaluation of blood immune cells. Representation of standardized values (z-scores) from the percentages of the main innate [eosinophils (A), total NK cells, (B) Ly6C^{+} NK cells (C), neutrophils (D), and monocytes Ly6C^{high} (E)] and adaptive immune cell populations over time [total B cells (F), total T cells (G), CD4^{+} T cells (H), CD8^{+} T cells (L), and activation compartments

within CD4+ and CD8+ T cells:: CD62L$^-$ (I and M, respectively), CD62L^{high} (J and N, respectively) and CD62L^{int} (K and O, respectively). The representation combines the results from the 3 independent experimental sets and z-scores were calculated based on the mean and standard deviation of all time points combined for each set independently. Each dot represents one animal, where males are depicted as teal diamonds and females as orange circles. Lines represent the best fit equation representative of the data [either linear regression or second order polynomial (quadratic) functions].

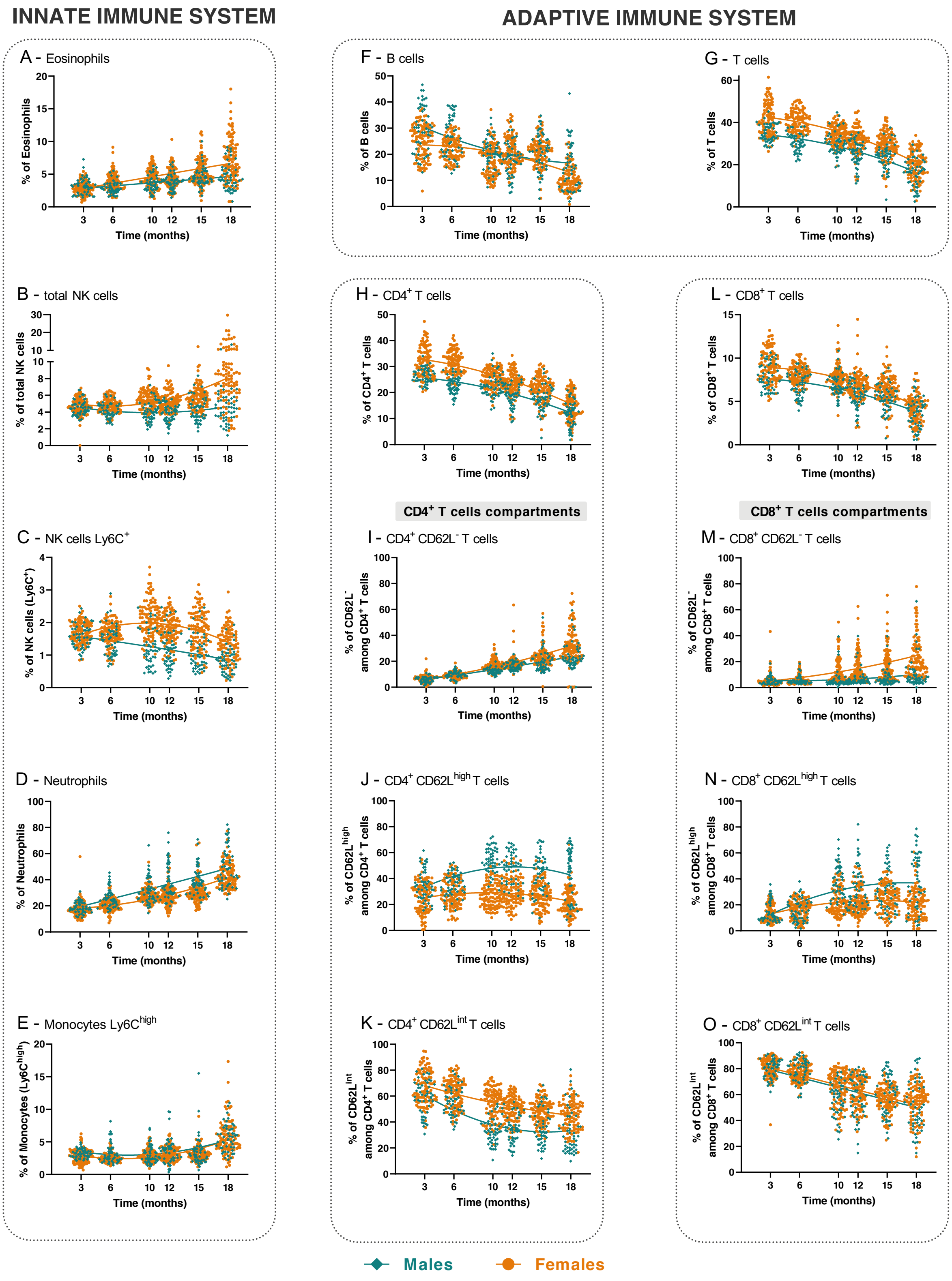

Supplementary Figure 6. Longitudinal evaluation of blood immune cells of survivors. Percentages of the main innate [eosinophils (A), total NK cells, (B) Ly6C^{+} NK cells (C), neutrophils (D), and monocytes Ly6C^{high} (E)] and adaptive immune cell populations over time [total B cells (F), total T cells (G), CD4^{+} T cells (H), CD8^{+} T cells (L), and activation compartments within CD4+ and CD8+ T cells:: CD62L^{-} (I and M,

respectively), CD62L^{high} (J and N, respectively) and CD62L^{int} (K and O, respectively). The representation combines the results from the 3 independent experimental sets. Each dot represents one animal, where males are depicted as teal diamonds and females as orange circles. Lines represent the best fit equation representative of the data [either linear regression or second order polynomial (quadratic) functions].

Supplementary Table 1. Number of mice analyzed at each timepoint for each independent experiment/set.

Timepoint (months)	Set1		Set2		Set3		TOTAL		
	Males	Females	Males	Females	Males	Females	Males	Females	Both
3	35	34	35	35	39	40	109	109	218
6	35	35	35	35	35	39	105	109	214
10	31	35	35	34	32	39	98	108	206
12	24	32	31	33	26	38	81	103	184
15	22	32	25	31	20	38	67	101	168
18	15	31	18	29	15	37	48	97	145

Supplementary Table 2. Linear mixed models testing the effect of time (aging) and sex on the percentages of $LY6C^-$ NK cells.

Dependent variable	Parameters	Estimate (B)	SE	p-value[b]	95% CI Lower Bound	95% CI Upper Bound
**$Ly6C^-$ NK cells**	Intercept	3,544	0,062	**<0,001**	3,422	3,666
	cTime	0,130	0,014	**<0,001**	0,103	0,157
	Sex[a]	-0,761	0,090	**<0,001**	-0,937	-0,584
	cTime * Sex[a]	-0,099	0,021	**<0,001**	-0,142	-0,057
	$cTime^2$	0,009	0,002	**<0,001**	0,005	0,014
	$cTime^2$ * Sex[a]	-0,001	0,003	0,707	-0,008	0,005

Supplementary Table 3. Linear mixed models testing the effect of time (aging) and sex on the percentages of the blood main innate immune cells, controlling for experimental set differences.

Dependent variable	Parameters	Estimate (B)	SE	p-value[b]	95% CI	
					Lower Bound	Upper Bound
**Eosinophils**	Intercept	4.178	0.118	**<0.001**	3.945	4.410
	cTime	0.227	0.018	**<0.001**	0.192	0.261
	Sex[a]	-0.902	0.142	**<0.001**	-1.180	-0.623
	cTime * Sex[a]	-0.100	0.028	**<0.001**	-0.156	-0.045
	$cTime^2$	0.002	0.003	0.493	-0.004	0.008
	$cTime^2$ * Sex[a]	0.005	0.005	0.281	-0.004	0.014
	Set2 vs others	0.828	0.114	**<0.001**	0.603	1.053
	Set3 vs others	0.376	0.112	**0.001**	0.155	0.597
**Total NK cells**	Intercept	5,221	0,107	**<0,001**	5,011	5,431
	cTime	0,138	0,016	**<0,001**	0,106	0,170
	Sex[a]	-1,413	0,125	**<0,001**	-1,658	-1,167
	cTime * Sex[a]	-0,147	0,025	**<0,001**	-0,196	-0,097
	$cTime^2$	0,007	0,003	**0,011**	0,002	0,012
	$cTime^2$ * Sex[a]	0,003	0,004	0,471	-0,005	0,011
	Set2 vs others	0,341	0,107	**0,002**	0,129	0,553
	Set3 vs others	0,214	0,106	**0,044**	0,006	0,422
**$Ly6C^+$ NK cells**	Intercept	1.901	0.049	**<0.001**	1.805	1.997
	cTime	-0.017	0.004	**<0.001**	-0.024	-0.009
	Sex[a]	-0.683	0.054	**<0.001**	-0.789	-0.577
	cTime * Sex[a]	-0.032	0.006	**<0.001**	-0.044	-0.020
	$cTime^2$	-0.007	0.001	**<0.001**	-0.008	-0.006
	$cTime^2$ * Sex[a]	0.007	0.001	**<0.001**	0.005	0.009
	Set2 vs others	0.065	0.054	0.229	-0.041	0.170
	Set3 vs others	-0.026	0.052	0.622	-0.129	0.077
**Neutrophils**	Intercept	24.734	0.742	**<0.001**	23.275	26.193
	cTime	1.638	0.075	**<0.001**	1.490	1.787
	Sex[a]	8.481	0.909	**<0.001**	6.692	10.271
	cTime * Sex[a]	0.312	0.123	**0.012**	0.069	0.555
	$cTime^2$	0.056	0.013	**<0.001**	0.030	0.081
	$cTime^2$ * Sex[a]	-0.083	0.020	**<0.001**	-0.122	-0.043
	Set2 vs others	1.907	0.673	**0.005**	0.580	3.233
	Set3 vs others	4.788	0.658	**<0.001**	3.491	6.085

**Monocytes ($Ly6C^{high}$)**	Intercept	3.157	0.117	**<0.001**	2.927	3.387
	cTime	0.173	0.067	**0.010**	0.041	0.305
	Sex[a]	0.592	0.121	**<0.001**	0.354	0.830
	cTime * Sex[a]	-0.060	0.095	0.526	-0.247	0.126
	$cTime^2$	0.024	0.001	**<0.001**	0.021	0.027
	$cTime^2$ * Sex[a]	-0.009	0.002	**<0.001**	-0.014	-0.005
	Set2 vs others	-0.379	0.138	**0.007**	-0.652	-0.105
	Set3 vs others	-0.726	0.137	**<0.001**	-0.997	-0.455

[a] Reference category is female;
[b] Statistically significant results (p-value < 0.05) are highlighted in bold;
CI (confidence interval); SE (standard error).

Supplementary Table 4. Linear mixed models testing the effect of time (aging) and sex on the percentages of the blood main adaptive immune cells, controlling for experimental set differences.

Dependent variable	Parameters	Estimate (B)	SE	p-value[b]	95% CI	
					Lower Bound	Upper Bound
**B cells**	Intercept	24.319	0.530	**<0.001**	23.277	25.362
	cTime	-0.793	0.046	**<0.001**	-0.884	-0.703
	Sex[a]	-1.137	0.603	0.060	-2.323	0.050
	cTime * Sex[a]	-0.156	0.074	**0.035**	-0.301	-0.011
	$cTime^2$	-0.051	0.010	**<0.001**	-0.070	-0.032
	$cTime^2$ * Sex[a]	0.110	0.015	**<0.001**	0.080	0.139
	Set2 vs others	-3.603	0.565	**<0.001**	-4.718	-2.488
	Set3 vs others	-6.773	0.557	**<0.001**	-7.870	-5.675
**T cells**	Intercept	33.269	0.548	**<0.001**	32.190	34.347
	cTime	-1.472	0.052	**<0.001**	-1.575	-1.369
	Sex[a]	-6.969	0.619	**<0.001**	-8.189	-5.750
	cTime * Sex[a]	0.304	0.083	**<0.001**	0.141	0.467
	$cTime^2$	-0.058	0.009	**<0.001**	-0.076	-0.040
	$cTime^2$ * Sex[a]	0.018	0.014	0.188	-0.009	0.046
	Set2 vs others	1.521	0.586	**0.010**	0.366	2.675
	Set3 vs others	1.846	0.576	**0.002**	0.710	2.982
**$CD4^+$ T cells**	Intercept	25.317	0.428	**<0.001**	24.475	26.159
	cTime	-1.171	0.039	**<0.001**	-1.248	-1.094
	Sex[a]	-5.369	0.476	**<0.001**	-6.305	-4.432
	cTime * Sex[a]	0.270	0.061	**<0.001**	0.149	0.391
	$cTime^2$	-0.037	0.007	**<0.001**	-0.050	-0.023
	$cTime^2$ * Sex[a]	0.010	0.011	0.335	-0.011	0.031
	Set2 vs others	0.478	0.468	0.308	-0.444	1.401
	Set3 vs others	0.882	0.460	0.057	-0.025	1.790
**$CD62L^-$ among $CD4^+$ T cells**	Intercept	15.787	0.431	**<0.001**	14.937	16.636
	cTime	1.800	0.072	**<0.001**	1.659	1.942
	Sex[a]	-2.461	0.572	**<0.001**	-3.589	-1.334
	cTime * Sex[a]	-0.759	0.112	**<0.001**	-0.980	-0.538
	$cTime^2$	0.054	0.009	**<0.001**	0.036	0.071
	$cTime^2$ * Sex[a]	-0.055	0.014	**<0.001**	-0.082	-0.027
	Set2 vs others	0.732	0.292	**0.013**	0.156	1.308
	Set3 vs others	2.928	0.287	**<0.001**	2.363	3.494

**CD62L^{int} among CD4$^+$ T cells**	Intercept	57.077	0.920	**<0.001**	55.270	58.885
	cTime	-1.536	0.101	**<0.001**	-1.735	-1.336
	Sex[a]	-16.109	1.112	**<0.001**	-18.293	-13.925
	cTime * Sex[a]	-0.005	0.158	0.975	-0.318	0.307
	cTime^2	0.044	0.020	**0.029**	0.004	0.084
	cTime^2 * Sex[a]	0.117	0.031	**<0.001**	0.056	0.178
	Set2 vs others	0.400	0.902	0.658	-1.379	2.180
	Set3 vs others	-9.440	0.889	**<0.001**	-11.193	-7.686
**CD62L^{hi} among CD4$^+$ T cells**	Intercept	26.643	0.992	**<0.001**	24.692	28.595
	cTime	-0.217	0.115	0.061	-0.444	0.011
	Sex[a]	18.920	1.200	**<0.001**	16.560	21.280
	cTime * Sex[a]	0.802	0.179	**<0.001**	0.448	1.155
	cTime^2	-0.093	0.018	**<0.001**	-0.129	-0.058
	cTime^2 * Sex[a]	-0.065	0.028	**0.021**	-0.120	-0.010
	Set2 vs others	-1.839	0.958	0.057	-3.730	0.053
	Set3 vs others	7.931	0.944	**<0.001**	6.068	9.794
**CD8$^+$ T cells**	Intercept	7.113	0.134	**<0.001**	6.850	7.377
	cTime	-0.297	0.036	**<0.001**	-0.368	-0.225
	Sex[a]	-1.393	0.160	**<0.001**	-1.708	-1.078
	cTime * Sex[a]	0.032	0.052	0.541	-0.070	0.134
	cTime^2	-0.021	0.002	**<0.001**	-0.025	-0.016
	cTime^2 * Sex[a]	0.011	0.003	**0.001**	0.005	0.018
	Set2 vs others	0.470	0.130	**<0.001**	0.215	0.725
	Set3 vs others	0.486	0.128	**<0.001**	0.235	0.737
**CD62L$^-$ among CD8$^+$ T cells**	Intercept	13.644	0.673	**<0.001**	12.321	14.967
	cTime	1.370	0.390	**<0.001**	0.606	2.134
	Sex[a]	-6.212	0.694	**<0.001**	-7.577	-4.847
	cTime * Sex[a]	-1.133	0.549	**0.039**	-2.210	-0.056
	cTime^2	0.042	0.008	**<0.001**	0.027	0.057
	cTime^2 * Sex[a]	-0.016	0.013	0.199	-0.041	0.009
	Set2 vs others	0.904	0.800	0.260	-0.671	2.478
	Set3 vs others	-3.087	0.794	**<0.001**	-4.649	-1.525
**CD62L^{int} among CD8$^+$ T cells**	Intercept	69.898	0.975	**<0.001**	67.982	71.815
	cTime	-1.965	0.109	**<0.001**	-2.181	-1.749
	Sex[a]	-0.945	1.239	0.446	-3.380	1.490
	cTime * Sex[a]	0.086	0.172	0.616	-0.254	0.427
	cTime^2	0.029	0.019	0.119	-0.008	0.066
	cTime^2 * Sex[a]	-0.008	0.029	0.781	-0.064	0.048
	Set2 vs others	-4.366	0.817	**<0.001**	-5.981	-2.751
	Set3 vs others	-7.901	0.804	**<0.001**	-9.489	-6.313

**CD62L^{hi} among CD8^{+} T cells**	Intercept	16.737	0.947	**<0.001**	14.875	18.600
	cTime	0.587	0.103	**<0.001**	0.385	0.790
	Sex[a]	6.507	1.207	**<0.001**	4.136	8.877
	cTime * Sex[a]	1.035	0.163	**<0.001**	0.715	1.355
	$cTime^2$	-0.087	0.017	**<0.001**	-0.121	-0.053
	$cTime^2$ * Sex[a]	0.038	0.027	0.152	-0.014	0.091
	Set2 vs others	4.735	0.782	**<0.001**	3.175	6.295
	Set3 vs others	11.035	0.769	**<0.001**	9.502	12.568

[a] Reference category is female;
[b] Statistically significant results (p-value < 0.05) are highlighted in bold;
CI (confidence interval); SE (standard error).
